# Conditional QTL mapping for seed germination and seedling traits under salt stress and candidate gene prediction in wheat

**DOI:** 10.1038/s41598-022-25703-3

**Published:** 2022-12-05

**Authors:** Xin Guo, Chongning Wu, Dehua Wang, Guanying Wang, Kaituo Jin, Yingjie Zhao, Jichun Tian, Zhiying Deng

**Affiliations:** 1 State Key Laboratory of Crop Biology, Key Laboratory of Crop Biology of Shandong Province, Group of Wheat Quality Breeding, Agronomy College, Shandong Agricultural University, Tai’an, Shandong People’s Republic of China; 2 Taiyuan Agro-Tech Extension and Service Center, 030000 Taiyuan, Shanxi People’s Republic of China

**Keywords:** Genetics, Plant sciences

## Abstract

Breeding new wheat varieties with salt resistance is one of the best ways to solve a constraint on the sustainability and expansion of wheat cultivation. Therefore, understanding the molecular components or genes related to salt tolerance must contribute to the cultivation of salt-tolerant varieties. The present study used a recombinant inbred line (RIL) population to genetically dissect the effects of different salt stress concentrations on wheat seed germination and seedling traits using two quantitative trait locus (QTL) mapping methods. A total of 31 unconditional and 11 conditional QTLs for salt tolerance were identified on 11 chromosomes explaining phenotypic variation (PVE) ranging from 2.01 to 65.76%. Of these, 15 major QTLs were found accounting for more than 10% PVE. QTL clusters were detected on chromosomes 2A and 3B in the marker intervals ‘wPt-8328 and wPt-2087’ and ‘wPt-666008 and wPt-3620’, respectively, involving more than one salt tolerance trait. QRdw3B and QSfw3B.2 were most consistent in two or more salt stress treatments. 16 candidate genes associated with salt tolerance were predicted in wheat. These results could be useful to improve salt tolerance by marker-assisted selection (MAS) and shed new light on understanding the genetic basis of salt tolerance in wheat.

## Introduction

The degree of land salinization has a significant upward trend in the world, causing serious harm to the quality and yield of crops^1^. To date, the arable land affected by salinity worldwide is greater than 800 million ha, which is approximately six percent of the global land area^2,3^. Moreover, the proportion of salt-affected soil seems to be increased because of irrigation every year, which could result in salt accumulation in agricultural soils^4,5^. Therefore, salinization has become a major limitation of grain yield^2^. Wheat (*Triticum aestivum* L*.*) is one of the most important food crops in the world, and it is a part of the daily diet of over 70% of the world population^6^. To meet the food needs of the growing world population, a significant increase in wheat yield is required^7,8^. However, most wheat cultivars hardly have salt tolerance in production because salinity stress influences seedling establishment at early growth stages of common wheat and severely reduces yield^9^. Therefore, breeding salt-tolerant wheat cultivars is a practical method to reduce the harm of salinization for food security^10^.

From genetic and physiological points of view, salt tolerance is complex. Tolerance often shows the characteristics of a multigenic trait controlled by polygenes^11^. Quantitative trait locus (QTL) mapping provides an effective approach to dissect complicated quantitative traits into component loci to study their relative effects on a specific trait^12,13^, thereby providing breeders with targets for marker-assisted selection (MAS)^14^. Previous researchers have identified some QTLs for salt tolerance traits in some crops, such as rice (*Oryza sativa*)^15^ and maize (*Zea mays*)^16^*.* Using common wheat, some results were also reported by QTL mapping^17–21^ and genome-wide association studies (GWAS) under salt stress conditions^21–23^.

Salinity stress negatively affects plant growth, development and overall productivity by inducing ion toxicity, osmotic stress, hormonal disturbance and oxidative stress^24,25^. At the initial stage of salt stress, the water absorption capacity of the root system is reduced, and water loss from the leaves is accelerated due to osmotic stress of high salt accumulation in soil and plants^26^. Over time, soil salinity causes toxic concentrations of Na^+^ to accumulate in leaves. This imposes an additional limitation to growth by reducing the longevity of photosynthetic tissues^27^.

There were two pathways that play a key role in salt tolerance in wheat, namely, *HKT* genes that mediate Na+ exclusion and the *SRO* gene that regulates ROS homeostasis^2^. *TmHKT1;4-A2* decreased the leaf blade Na+ concentration by 50%, *TmHKT1;5-A* decreased it by 30%, and both genes together decreased it by 60%^28^. *TaSRO* can act as a transcription factor regulator or covalent binding factor to interact with different transcription factors, thereby extensively participating in plant responses to stress conditions^29^. In addition, many other genes have been investigated. For example: *TaAQP8*, *NHX* gene family, *TaCYP81D5*, *TaCIPK29*, etc^30–34^.

Generally, previous studies evaluated characteristics under salt stress in wheat, mainly including the germination rate (GR), germination potential (GP), mean root length (MRL), root fresh weight (RFW), root dry weight (RDW), shoot height (SH), shoot fresh weight (SFW) and shoot dry weight (SDW), at the seedling stage^17–19,22,35^*.* Few studies have been reported at the adult stage involving plant height, spike length, grain number, yield per plant, spike number, and thousand-kernel weight^20,23^. Because QTL mapping based on linkage and marker trait association can be effectively used for gene pyramiding, germplasm screening of diversified material for abiotic (salinity, cold, salt, drought) and biotic stresses (disease, pest), etc., the identification and location of specific genes mediating quantitative characters is of great importance in plant breeding^54^. The objectives of QTL mapping are to offer a direct means to investigate the number of genes influencing the trait, to determine the location of the gene that affects traits of interest, to know the effect of genes on variation of the trait and to carry out a study on linkage between genes of interest^54^. To date, more than 500 QTLs (excluding those involved in digenic epistatic interactions and QTL x treatment interactions) have been identified on all 21 wheat chromosomes, explaining 8.4% to 40.0% of the phenotypic variation for each QTL^36^. Only a dozen significant QTLs, mostly distributed on chromosomes 2D, 3B, 4B, 4D, 5A, and 7A^25^, were reported. For example, a locus Kna 1 for Na^+^ exclusion was mapped on chromosome 4DL^37^, and the QTL QNax.aww-7AS explaining 40% of Na^+^ variation was identified using two mapping populations^38^. For shoot dry weight, there was one major QTL, qSNAX.7A.3, with approximately 19% phenotypic variation^19^. Most 31 QTLs for salinity tolerance were detected on chromosomes 3B and 5B, while two QTLs for fresh weight and height of shoots were detected on chromosomes 1A and 3A, which explained 18% and 12.9% of the phenotypic variation, respectively^17^. There were some QTLs for the ionic traits across the three growth stages on 1BS, 2AL, 2BS and 3AL detected^22^. Two QTLs for seedling height and five QTLs for taproot length were detected using 168 doubled haploid lines^39^. 69 QTLs associated with seven seedling traits were found on 20 chromosomes, except for chromosome 1A^40^.

Although many QTLs for wheat salt tolerance traits have been identified, some of them are not well used in wheat breeding under salt stress, and they were mapped using the unconditional QTL mapping method, which could not dissect the genetic difference under salt stress with different salt concentrations. To dissect the interactions and molecular differences under different treatments or between related traits, the method of conditional QTL mapping was developed by Zhu^41^, which can be used to exclude the contribution of a treatment from the variation of the resultant treatment. Conditional variation or net variation is defined as the remaining variation of the resultant treatment, suggesting that the extra effects of these genes/QTLs are independent of the causal treatment. Therefore, the genetic effects of conditional variations of the resultant treatment can be dissected at the QTL/gene level. By comparing unconditional and conditional QTLs, the genetic interdependencies between them can be identified at the individual QTL level. This comparison might provide valuable information for marker-assisted selection to improve salt tolerance without negative effects on wheat.

To date, conditional QTL mapping has rarely been used in wheat salt stress for traits related to seed germination and seedling stage. Therefore, a recombinant inbred line (RIL) population derived from the cross between Nuomai 1 (NM 1) and Gaocheng 8901 (GC 8901) was used to genetically dissect the effects of different salt stress concentrations on wheat seed germination and seedling traits using unconditional and conditional quantitative trait locus (QTL) mapping. The objectives of this study were to (1) identify novel stable QTLs under various salt concentrations using unconditional and conditional QTL mapping and (2) find new candidate genes for significant QTLs that were predicted. These results will provide important information on breeding wheat cultivars with salt tolerance.

## Results

### Phenotypic variation and correlation analysis

All of the evaluated traits showed approximately continuous variation in each of the treatments (Tables 1 and 2). The salt-tolerant parent NM1 showed significantly higher values (14.46–50% greater) than the salt-sensitive parent GC8901 for GR in the T1 and T2 treatments. There were no significant differences in GR in the two parents under the N treatment. However, for GP, significant differences were not found in the two parents under the N, T1 and T2 treatments (Table 1). With increasing salt concentration, the means of GR and GP decreased. In Table 2, significant differences were found between the two parents for seedling traits under different treatments, such as MRL under the N, T1 and T2 treatments, RFW under the T3 treatment, and SDW under the T1, T2 and T3 treatments. In the RIL population, transgressive segregation was observed on both the high and low sides for GR, MRL, RFW, RDW, SH, SFW and SDW, which indicated that the alleles with positive effects were contributed by both parents (Tables 1 and 2).

**Table 1:** Phenotypic performance for germination rate and germination potential of the two parents and RIL population under different salt treatments.

Traits	Treatment	Parent		RILs				
		NM1	GC 8901	Mean	Range	S.D	Skewness	Kurtosis
GR	N	0.99	0.99	0.80	0.46–1.00	0.11	− 0.873	0.699
	T1	0.95*	0.84	0.59	0.10–0.92	0.14	− 0.211	− 0.156
	T2	0.57*	0.38	0.40	0.10–0.92	0.16	0.408	− 0.062
GP	N	0.49	0.52	0.42	0.10–0.84	0.15	0.269	− 0.271
	T1	0.23	0.19	0.22	0.02–0.60	0.11	0.778	0.577
	T2	0.10	0.11	0.11	0.00–0.42	0.09	0.815	0.620

GR, germination rate; GP, germination potential; N, 0 mM NaCl; T1, 50 mM NaCl; T2, 100 mM NaCl; S.D., standard error of the mean.

* and **Means significant at the 0.05 and 0.01 probability levels, respectively.

**Table 2:** Phenotypic performance for traits related to seeding growth of the two parents and RIL population under different salt treatments.

Traits	Treatment	Parent		RILs				
		NM1	GC8901	Mean	Range	S.D.	Skewness	Kurtosis
MRL (cm per plant)	N	5.53	9.32**	7.80	2.02–18.37	3.45	0.26	− 0.56
	T1	6.65	8.20**	7.97	1.53–15.77	3.27	-0.20	− 0.93
	T2	2.53	4.10**	6.80	0.23–14.57	2.98	-0.03	− 0.37
	T3	3.33	2.50	3.44	0.70–12.42	1.95	1.13	1.37
RFW (g per plant)	N	0.25	0.20	0.25	0.05–0.95	0.11	− 1.58	0.01
	T1	0.25	0.27	0.23	0.03–0.62	0.09	0.80	0.21
	T2	0.12	0.17**	0.22	0.01–0.77	0.10	1.51	0.77
	T3	0.15*	0.08	0.17	0.02–1.82	0.15	− 0.86	− 0.93
RDW (g per plant)	N	0.04	0.03	0.03	0.01–0.18	0.02	0.78	0.19
	T1	0.04	0.05*	0.03	0.01–0.11	0.02	− 0.40	0.64
	T2	0.03**	0.01	0.04	0.01–0.18	0.03	0.48	0.23
	T3	0.02**	0.01	0.04	0.01–0.18	0.03	0.49	0.63
SH (cm per plant)	N	13.53**	11.17	13.91	8.60–19.53	1.92	0.02	0.14
	T1	13.35**	11.13	13.18	6.90–18.52	2.47	− 0.28	− 0.64
	T2	10.35	10.30	12.54	4.68–23.97	3.18	− 0.24	− 0.10
	T3	8.95**	7.00	8.86	1.83–15.68	2.99	− 0.17	− 0.70
SFW (g per plant)	N	0.62**	0.52	0.68	0.11–2.60	0.28	1.86	1.00
	T1	0.49	0.47	0.65	0.07–1.33	0.25	0.31	− 0.25
	T2	0.40*	0.35	0.59	0.04–1.65	0.26	0.43	0.50
	T3	0.34	0.32	0.41	0.04–1.84	0.21	0.13	0.25
SDW (g per plant)	N	0.07	0.07	0.07	0.03–0.73	0.05	0.13	0.20
	T1	0.07*	0.04	0.06	0.01–0.10	0.01	0.04	1.17
	T2	0.07*	0.03	0.06	0.03–0.80	0.07	0.11	0.12
	T3	0.05**	0.02	0.06	0.01–0.47	0.04	0.85	0.51

MRL Main root length; RFW Root fresh weight; RDW Root dry weight; SH Seeding height; SFW Seeding fresh weight; SDW Seeding dry weight; N, T1, T2 and T3 were 0, 50, 100 and 200 mM NaCl, respectively.

* and **Means significant at the 0.05 and 0.01 probability levels, respectively.

Significant positive correlations were observed between SH and MRL and SFW and RFW under all treatments, but they were negatively correlated with RDW (Table S1). The highest positive correlation coefficient was observed with 0.8 between SH and MRL, but the lowest negative correlation coefficient was − 0.59 between SH and RDW under the T2 treatment. MRL was negatively correlated with RDW under all treatments but positively correlated with SDW, SFW, RFW and GR under most treatments. SFW was significantly and negatively correlated with RDW but positively correlated with SH, MRL, SDW, SFW, RDW, RFW and GR. RDW was significantly and positively correlated with SDW under the N, T2 and T3 treatments but negatively correlated with all other traits except GP under most of the treatments. In most of the different salt stress treatments, GP was significantly and positively correlated with GR, and GR was significantly and positively correlated with SH, MRL, SFW and GP.

### QTL analysis

A total of 31 unconditional QTLs and 11 conditional QTLs for 8 traits were detected on nine chromosomes (Tables 3, 4 and 5). Of 31 unconditional QTLs, 11 unconditional QTLs for GR and GP were identified, explaining from 2.1 to 35.34% of phenotypic variation (PVE) (Table 3), and twenty unconditional QTLs were found for SH, MRL, SDW, SFW, RDW and RFW, accounting for 4.58–65.76% of the phenotypic variation (Table 4). Fourteen major unconditional QTLs were identified. The conditional QTLs accounted for 1.07–20.09% of the phenotypic variation under the different treatments (Table 5).

**Table 3:** QTLs with additive effects (Add) detected at the seedling stage under the N, T1 and T2 conditions.

Trait	Treatment	QTL	Position (cM)	Marker interval	LOD	PVE (%)	Add
GP	N	QGp6A.2	60	wPt-3524–wPt-5652	2.72	3.09	− 0.03
	T1	QGp2B	53	wPt-9336–wPt-7350	2.88	5.13	0.03
	T1	QGp6A.1	22	wPt-3091–wPt-0959	2.6	3.55	− 0.02
	T2	QGp3B	160	wPt-666008–wPt-5870	6.97	35.34	− 0.4
GR	N	QGr3B.1	166	wPt-3620–wPt-7906	2.62	4.9	0.04
	N	QGr6B	288	wPt-730273–wPt-6329	3.52	6.4	0.03
	T1	QGr3A	152	wPt-8876–wPt-730156	2.76	3.88	0.03
	T1	QGr5A	4	wPt-1903–wPt-3069	2.57	2.64	0.02
	T1	QGr6A	22	wPt-0959–wPt-666988	2.59	2.7	− 0.02
	T2	QGr2A	247	wPt-9951–wPt-2273	2.64	2.29	0.04
	T2	QGr3D	163	wPt-730115–wPt731146	2.65	2.1	− 0.02

N, T1, T2 and T3 are the same as in Table 2.

**Table 4:** QTLs with additive effects of traits related to seedling growth detected in the RILs under the N, T1, T2 and T3 conditions.

Traits	Treatment	QTL	Position (cM)	Marker interval	LOD	PVE (%)	Add
MRL	N	QMrl2B	88	wPt-1454–wPt-4559	2.98	5.89	0.85
	T1	QMrl4A	152	wPt-672107–wPt-664749	2.70	4.90	0.72
	T2	QMrl3B.1	164	wPt-5870–wPt-3620	2.53	7.00	− 2.01
	T3	QMrl2A	198	wPt-2185–wPt-7187	3.02	30.18	1.40
	T3	QMrl3B.2	182	wPt-7526–wPt-5072	4.62	11.07	− 0.79
RFW	T3	QRfw2A	215	wPt-6207–wPt-2087	3.66	14.89	0.12
RDW	N	QRdw3B.1	160	wPt-666008–wPt-5870	10.38	45.68	− 0.03
	N	QRdw3B.1	160	wPt-666008–wPt-5870	7.83	34.71	− 0.04
	T1	QRdw3B.1	160	wPt-666008–wPt-5870	9.58	55.78	− 0.02
	T2	QRdw3B.1	160	wPt-666008–wPt-5870	13.56	45.00	− 0.05
	T3	QRdw3B.1	160	wPt-666008–wPt-5870	25.83	65.76	− 0.05
	T3	QRdw3B.2	164	wPt-5870–wPt-3620	14.83	56.59	− 0.05
SH	N	QSh4A	155	wPt-6404–wPt-2291	2.58	4.84	0.42
	T1	QSh2A	213	wPt-2185–wPt-7187	2.91	5.34	1.40
	T3	QSh2A	213	wPt-2185–wPt-7187	3.17	9.27	1.26
SFW	N	QSfw2A	194	wPt-2185–wPt-7187	3.02	15.99	0.15
	N	QSfw3B.1	160	wPt-666008–wPt-5870	5.17	23.63	− 0.30
	N	QSfw3B.2	164	wPt-5870–wPt-3620	4.15	16.51	− 0. 28
	T2	QSfw3B.2	164	wPt-5870–wPt-3620	2.55	4.58	− 0.11
SDW	T3	QSdw3B	164	wPt-5870–wPt-3620	32.80	53.67	− 0.16

N, T1, T2 and T3 are the same as in Table 2.

**Table 5:** Additive effects of conditional QTLs in the RIL population.

Trait	QTL	Position	Marker interval	LOD	PVE(%)	Add
MRL	QMrl2A(T2|N)	156	wPt-664128–wPt-5647	3.62	6.40	− 0.62
	QMrl3B(T2|T1)	169	wPt-7906–wPt-2559	3.45	6.29	− 1.13
	QMrl2A(T3|T2)	208	wPt-8328–wPt-2185	3.81	8.51	0.82
SH	QSh2A(T2|T1)	212	wPt-8328–wPt-2185	4.19	7.45	1.24
SFW	QSfw3B(T2|N)	162	wPt-666008–wPt-5870	6.79	1.09	0.53
	QSfw3B(T2|N)	164	wPt-5870–wPt-3620	8.58	1.07	0.53
	QSfw3B(T2|T1)	164	wPt-5870–wPt-3620	7.16	3.39	− 0.51
	QSfw3B(T3|T2)	167	wPt-3620–wPt-7906	3.00	5.33	0.50
RDW	QRdw2B(T1|N)	82	wPt-0047–wPt-1454	2.55	4.90	− 0.01
RFW	QRfw2A(T3|T2)	221	wPt-3896–wPt-2644	8.69	4.50	0.78
SDW	QSdw3B(T2|T1)	163	wPt-666008–wPt-5870	13.3	20.09	− 0.35

**Figure 1:**
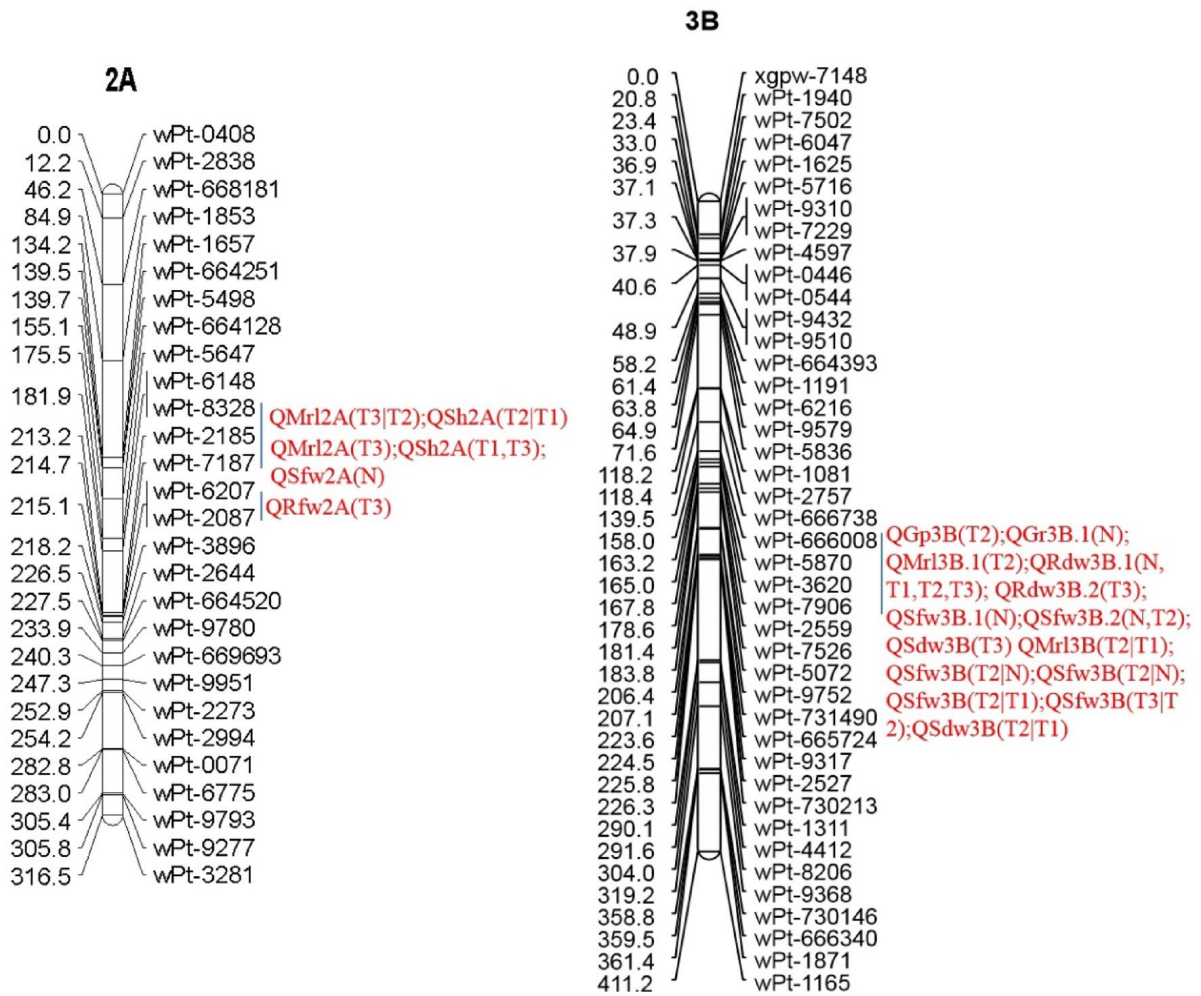
QTL clusters on the genetic map including unconditional and conditional QTLs under salt stress at different concentrations.

Four unconditional QTLs were detected for GP in three different concentrations of salt stress (Table 3), which were distributed on chromosomes 2B, 3B, and 6A. The additive effect of QGp3B came from GC 8901 with a maximum PVE of 35.34%, which was co-located with the unconditional QTLs QRdw3B*,* QSfw3B.1 and QSdw3B (Table 4, Fig. 1).

Seven unconditional QTLs were detected for GR under salt stress at three different concentrations (Table 3), accounting for from 2.1 to 6.4% of PVE, which were distributed on seven chromosomes (2A, 3A, 3B, 3D, 5A, 6A, 6B). The additive effect of two QTLs, QGr6A and QGr3D*,* came from GC 8901, and the other came from NM1. However, they all were minor QTLs. The QTL QGr3B.1 was colocalized with the other QTLs, QMrl3B.1, QRdw3B, QSfw3B.2 and QSdw3B.

Five unconditional QTLs and three conditional QTLs for the MRL were detected, and the PVE of a single QTL ranged from 4.90 to 30.18% (Tables 4 and 5). The synergistic alleles of the QTLs QMrl2A, QMrl2B and QMrl4A were derived from NM 1. The synergistic alleles of the QTLs QMrl3B.1 and QMrl3B.2 were derived from GC 8901. The unconditional QTL QMrl2A was co-located with the unconditional QTL QSh2A in the same marker interval. Three newly identified conditional QTLs were induced by salt stress. Of these, the conditional QTL QMrl2A(T3|T2) was located near the unconditional QTL QMrl2A and the conditional QTL QSh2A(T2/T1) with the same marker wPt-2185, which indicated that a locus on chromosome 2A may play an important role in seedling growth under salt stress.

One unconditional QTL and one conditional QTL were identified for RFW. The unconditional QTL QRfw2A was detected under the T3 treatment, and the PVE was 14.89% (Table 4). The conditional QTL identified for RFW was QRfw2A (T3|T2), which was induced by higher salt stress and played a critical role in salt tolerance*.*

Six unconditional QTLs and one conditional QTL controlling RDW were detected. The QTL QRdw3B.1 was identified in the same marker interval under four treatments, accounting for 34.7%-65.76% of PVE (Table 4). The synergistic alleles were derived from GC 8901. QRdw3B.2 was found under the T3 treatment. The conditional QTL QRdw2B(T1|N) was identified for RDW*,* which was induced by salt stress and may play a role under salt stress.

Three unconditional QTLs and one conditional QTL affecting SH were detected, and the PVE of each QTL ranged from 4.84 to 9.27%. The synergistic genes were all from NM 1. There were no QTLs detected under the T2 treatment. The conditional QTL QSh2A(T2|T1) identified for SH was induced by salt stress*,* which may play a role in salt tolerance.

Four unconditional QTLs and four conditional QTLs for the SFW were identified, ranging from 1.07 to 23.63% of PVE for a single QTL. Most of them were co-located with QRdw3B, except QSfw2A. Of the eight QTLs, the seven loci on chromosome 3B were derived from GC 8901. The QTL QSfw2A was contributed by NM 1-derived alleles. Four conditional QTLs were identified in almost the same marker intervals on chromosome 3B, and the unconditional QTLs for SFW under the N and T2 treatments were also found in similar marker intervals. This indicated that these loci on chromosome 3B seemed to be important for salt tolerance.

One unconditional QTL and one conditional QTL for SDW were observed with 53.67% and 20.09% PVE, respectively. Two QTLs were located on chromosome 3B in marker intervals wPt-5870-wPt-3620 and wPt-666008-wPt-5870 (Fig. 1). The synergistic allele was derived from GC 8901.

For biomass-related traits, two conditional QTLs were identified for different traits. The conditional QTLs QMrl2A(T3|T2), QSh2A (T2|T1) and QRfw2A(T3|T2) were located in the nearby marker intervals and near QMrl2A (Fig. 1). The QTLs QSfw3B(T2|N), QSfw3B(T2|N), QSfw3B(T2|T1), QSfw3B(T3|T2) and QSdw3B(T2|T1), QGr3B.1, QMrl3B.1, QRdw3B, QSfw3B.1 and QSfw3B.2 were located in the nearby marker intervals, and the latter was also identified in unconditional analysis for GR, MRL, RDW and SFW. However, the QTL QRdw2B(T1|N) was only detected by conditional QTL mapping.

### Prediction of candidate genes for important loci

Six markers with high PVE values were selected from loci significantly linked with the traits related to salt tolerance for prediction (Table S2). Some candidate genes were identified. The marker wPt-7187 on the chromosome had one candidate gene, *TraesCSU02G009300.1*, from wheat (Table S2). The functions of this gene are related to calcium ion binding and polysaccharide binding. Meanwhile, from *Oryza brachyantha*, one candidate gene, *OB01G45130*, was found to be related to the functions of peroxidase activity, calcium ion binding, and oxidoreductase activity, acting on NAD(P)H, with oxygen as an acceptor. It participates in the oxidation–reduction process. Four candidate genes were found for the marker wPt-2185 from wheat, but their functions are unknown. However, one candidate gene, *OB09G21770*, from *Oryza brachyantha* was found to be related to the functions calcium transmembrane transporter activity, phosphorylative mechanism, and calmodulin binding, which take part in the biological process of ion transport and calcium ion transport calcium ion transmembrane transport. This indicated that the four candidate genes perhaps had these functions but need to be further studied. For the marker wPt-2087, three candidate genes, *TraesCS2A02G048300*, *TraesCS2A02G048400*, and *TraesCS2A02G048500*, were found on chromosome 2A, but their functions were unknown in wheat. However, interestingly, each candidate gene was found in *Oryza brachyantha*, *Oryza glumipatula*, and *Brachypodium distachyon*. The functions of the OB06G15330 gene included ion channel activity and voltage-gated potassium channel activity, participating in the processes of ion transport, potassium ion transport, potassium ion transmembrane transport, and ion transmembrane transport. The *OGLUM01G14020* gene participated in the response to salt stress. For the marker wPt-5870, three and one candidate genes were found on chromosomes 3B and 2D, respectively, but the functions on chromosome 3B are unknown in wheat. The other eight homologous genes from *Oryza glumipatula*, *Aegilops tauschii*, *Oryza barthii*, *Triticum turgidum*, *Oryza brachyantha*, *Triticum dicoccoides*, *Oryza sativa Indica Group*, and *Oryza glaberrima* are all involved in the biological processes of sodium ion transport, transmembrane transport and response to salt stress. This indicated that the candidate genes from common wheat may be related to the response to salt stress, which needs to be further identified. For the marker wPt-3620, three candidate genes were found on chromosome 3B, but the functions are unknown in common wheat. However, in *Brachypodium distachyon*, the candidate gene *BRADI_4g16243v3* was related to the function of zinc ion binding and metal ion binding. For the marker wPt-666008, there were three candidate genes found on chromosomes 3B and 1D, but their functions are unknown. However, the candidate gene *ORGLA04G0060900* had the functions of magnesium ion binding and metal ion binding in *Oryza glaberrima*.

In general, these candidate genes found in common wheat may be related to salt tolerance, and their functions will be explored in future research.

## Discussion

Breeding salt-tolerant wheat varieties is an effective and environmentally sustainable way to utilize salt-affected soils and increase global food production^21,22^. Previous studies showed that most morphological and physiological indexes of wheat were differentially affected by salt stress, of which root dry weight, root fresh weight and the ratio of dry weight root to shoot were greatly affected and were also very sensitive to salt stress^55^. Similarly, the phenotypic performance of the present study also decreased with increasing salt concentration, but the germination rate and germination potential were greatly affected, which indicated that the salt tolerance of seed germination could also be important for wheat production. Although a number of physiological traits have been reported to correlate with salt tolerance in plants, the abilities of excluding Na^+^ and maintaining a high cytosolic K^+^/Na^+^ ratio seemed to be important for plant salt tolerance. In the present study, seedling biomass traits and germination traits were focused on salt stress. Shoot height was significantly positively correlated with shoot fresh weight under both non-salt stress and salt stress, which was almost consistent with previous studies^55,56^. In addition, Li found that the germination rate was significantly and positively correlated with shoot height and maximum root length, which was also found in our research. Meanwhile, the germination rate was also significantly and positively correlated with SFW and GP in the present study. These results indicated that germination traits were important for wheat growth under salt stress.

To speed up the breeding of salt-tolerant varieties by marker-assisted selection, knowledge of salt tolerance loci and genes is required. Previous researchers have studied many traits under salt stress with different concentrations at the seedling stage and adult stage using QTL mapping and GWAS^17–22,36^. Of these, some studies involved traits including Na^+^ exclusion/content, K^+^ content and K^+^/Na^+^ ratio, seedling shoot fresh weight, and plant height, which mainly mapped on chromosomes 2D, 3B, 4B, 4D, 7A and 5A for major QTLs (PVE > 20%)^36^. However, in the present study, the major QTLs were mainly involved in two chromosomes, 2A and 3B. On chromosome 3B, QTL clusters were found for shoot height, shoot fresh weight and shoot dry weight under salt stress in seedling stages^17^. Although the QTL loci were all found on chromosome 3B^36,43^, they were different loci by comparison with previous studies. Moreover, this new locus was involved in many traits, such as GP, MRL, RDW, SFW and SDW, under salt stress in this study, which was consistent with their significant phenotypic correlations. Therefore, it is important for salt tolerance and should be further studied in the future.

In addition, previous reports have identified some important QTLs or genes mainly affecting Na^+^ content, K^+^ content, and the K^+^/Na^+^ ratio on chromosome 2A. For example, the QTL_2AL.1 region (R^2^ > 16.93%) was associated with stress tolerance traits across three growth stages, germination, seedling and adult-field-grown plants, including the leaf K^+^/Na^+^ ratio, which is proximal to the codominant SSR marker gwm312, which is closely linked to the Nax1 gene^22^. By QTL mapping of NAX, there were two closely located QTLs, qSNAX.2A.1 and qSNAX.2A.1, and qRNAX.2A.1 coincided with the major NAX locus Nax1 or HKT1:4 in durum wheat and three NAX QTLs found on 2A in bread wheat^19,44,45^. In the present study, QTL clusters were also found on chromosome 2A in the regions between wPt-8328 and wPt-2087. These QTLs mainly involved salt tolerance traits, including MRL, SH and SFW, in the seedling stage, which was consistent with their significant positive phenotypic correlations. By comparing with the previous consensus map of different marker types, such as simple sequence repeat (SSR), diversity array technology (DArT) markers and SNP^46^, we found that this QTL cluster for seedling biomass should be different from the QTL_2AL.1 region; that is, this region perhaps involves a new salt tolerance gene, but which needs to be further studied in the future.

Although the conditional QTL mapping method has been used in previous studies^42,47^, it was not reported on the conditional QTL mapping of salt tolerance traits under different salt-stress conditions in common wheat. In fact, conditional QTL mapping is a good method to dissect QTLs/genes induced or not induced by salt stress. In this study, some QTLs were induced to be identified by salt stress, such as QRfw2A (T3|T2), QMrl2A(T2|N), QSh2A(T2|T1), and QMrl3B(T2|T1). Most interestingly, some of them were induced by low-salt stress conditions, and some were induced by high-stress conditions. Moreover, the QTLs controlling the SFW on chromosome 3B seemed to be unaffected by salt stress because they were all identified under different conditions. Therefore, its region is important for salt tolerance.

By screening the candidate genes for important loci on chromosomes 2A and 3B, there were a total of sixteen candidate genes, including *TraesCSU02G009300.1,* *TraesCSU02G212400*, *TraesCS2A02G048300*, *TraesCS3B03G0601400LC.1*, *TraesCS3B03G0301100LC.1*, and *TraesCS3B03G0143800LC* found in common wheat, but most of their molecular functions and biological processes are unknown, which are different from previous genes related to salt tolerance, such as *TaMYB32*, *TaOPR1*, *TaSRO1* and *TaHKT1*^48–50^. Of these, the predicted gene, *TraesCSU02G009300.1*, had the function of calcium ion binding and polysaccharide binding. However, most of the candidate genes from rice, *Brachypodium distachyon*, and *Triticum dicoccoides* in this study are related to the function of metal ion binding (calcium, zinc, sodium) and transmembrane transporter activity, mainly participating in sodium ion transport and response to salt stress. This indicates that these candidate genes predicted in common wheat are most likely related to salt tolerance, which should be further studied in the future.

## Conclusions

In all, a total of 31 unconditional QTLs and 11 conditional QTLs for 8 salt tolerance traits were detected on 11 chromosomes under different salt stresses. There were 14 major unconditional QTLs and one major conditional QTL identified. On chromosomes 2A and 3B, QTL clusters were found for salt tolerance traits. A total of sixteen candidate genes were predicted. This information is very useful in marker-assisted breeding to enhance salt tolerance in wheat.

## Materials and methods

### Ethics statement

All samples analysed in our study adhered to all local, national or international guidelines and legislation, and no ethical approval was required.

### Plant materials

The RIL population of 256 lines was developed by a single-seed descent method after crossing between Gaocheng 8901 (GC 8901) and Nuomai 1 (NM 1). The parent of NM 1 (Jiangsu Baihuomai/Guandong107) was bred by China Agricultural University and released in 2005 in Beijing. GC 8901 (77546-2/Linzhang) was bred by the Gaocheng Agricultural Science Research Institute and released in 1998 in Hebei Province. These materials were kept and provided by our team.

### Experimental design

The experiment was conducted in a hydroponic culture under a greenhouse at Shandong Agricultural University with a 16/8 day/night photoperiod, 27/20 °C day/night temperature and a relative humidity of approximately 60% in 2020. One hundred seeds for each parent and each RIL were selected. Seeds of parents and 256 RILs were sterilized in 3% H_2_O_2_ for 20 min, rinsed with deionized water, and then allowed to germinate on two-double filter paper in petri dishes containing distilled water in the dark at 20 ± 2 °C. They were evaluated for salt tolerance under four salt treatments, 0, 50, 100 and 200 mM NaCl, designated the N, T1, T2 and T3 treatments, respectively. Each treatment was designed with three replications. When the hypocotyl elongation was 1 cm, the most uniform seedlings were selected and placed in an 84-hole tray of 5 cm × 5 cm. Then, the seedling pan was placed in a culture pot with four salt concentrations. For each treatment, the phenotypic data of ten seedlings of each of the 256 lines were used for QTL analysis.

### Trait measurements

The germination rate (GR) and germination potential (GP) of wheat seeds were determined after treatment with four different salt concentrations for 4 and 7 days, respectively. Meanwhile, after 7 days of salt treatment, the ten most uniform seeding samples from each line and parents were collected and rinsed with distilled water. The roots and the shoots were separately harvested. Mean root length (MRL) and shoot height (SH) were recorded. The root fresh weight (RFW) and shoot fresh weight (SFW) were weighed, and then they were oven-dried at 103 °C for 1 h and then at 80 °C for 8 h. The root dry weight (RDW) and the shoot dry weight (SDW) were weighed.

### Statistical and QTL analysis

Statistical analyses (e.g., normal distribution and correlation) were performed using SPSS 17.0 software (SPSS, Chicago, USA). Trait measurements were averaged over three replications prior to QTL analysis.

The linkage map of the RIL population was constructed in a previous study^42^. A total of 501 markers were mapped, including 479 DArT markers, 17 SSR markers, 2 HMW-GS markers, and 3 Wx protein markers. It covered 4213.2 cM with an average distance of 8.4 cM, producing 25 linkage maps. These markers were identified on 21 chromosomes.

Conditional genetic analysis was conducted according to Deng et al.’s described method^42^. It was based on the phenotypic values under treatment 2 conditioned on treatment 1, which were obtained by the mixed-model approach^51^. Conditional phenotypic values y(T2|T1) were obtained by the mixed model approach for the conditional analysis of quantitative traits, where T2| T1 means treatment 2 conditioned on treatment 1. QGAStation 1.0^51^ software was used to calculate the conditional phenotypic values y(T1|T2), which were subsequently used as input data for conditional QTL mapping.

Unconditional and conditional QTL mapping were performed using the software QTL IciMapping V4.1^52^. The LOD threshold was set to 2.5. To clarify the designations of the examined QTLs, the following rules were adopted: ‘Q’ is an abbreviation of its corresponding trait, whereas a numerical number followed by an upper case letter, ‘A’, ‘B’, or ‘D’, is an indication of the chromosome number present in a given wheat genome where the corresponding QTL was detected, and if there is more than one QTL on one chromosome, a serial number behind a hyphen is added (e.g., QMrl3B.2 stands for the second QTL for MRL was detected on chromosome 3B).

### Forecasting candidate genes for salt tolerance at the seed germination and seedling stages

To identify the position of important QTL loci on a physical map and possible candidate genes, significant markers detected in this study were used to identify putative candidate genes according to the method of Ji^53^. A BLAST (Basic Local Alignment Search Tool) search was performed on the International Wheat Genome Sequencing Consortium database (wheat Chinese Spring IWGSC RefSeq v1.1 and v2.1 genome assembly; http://www.wheatgenome.org/) using the sequence of the significant DArT markers identified by QTL mapping. When a DArT marker sequence from the IWGSC was 100% identical to any wheat contig, the sequence was extended 2 Mb for each marker using the IWGSC BLAST results^53^. Then, the extended sequence was used to run a BLAST search at the National Center for Biotechnology Information (NCBI) database (http://www.ncbi.nlm.nih.gov) and Ensembl Plants (http://plants.ensembl.org/Triticum_aestivum/Tools/Blast) to confirm possible candidate genes and functions^53^.

### Ethics approval

Wheat is a common crop extensively cultivated worldwide. This study does not contain any research requiring ethical consent or approval.

## Supplementary Information

Supplementary Tables.

## Data Availability

All data used during the current study are included in this published article or are available from the corresponding author upon reasonable request.
