# Supplementary material for: Conditional QTL mapping for seed germination and seedling traits under salt stress and candidate gene prediction in wheat
Source: Sci Rep. 2022 Dec 5;12:21010. doi: 10.1038/s41598-022-25703-3 (PMC9722660; doi:10.1038/s41598-022-25703-3)
Supplement: Supplementary file 1 — Supplementary Tables. [file 41598_2022_25703_MOESM1_ESM.docx]

**Table S1 Correlation coefficients among traits under different salt stress conditions**

|  | | **SH** | | | | **MRL** | | | | **SDW** | | | | **SFW** | | | | **RDW** | | | | **RFW** | | | | **GR** | | | **GP** | | |
| --- | --- | --- | --- | --- | --- | --- | --- | --- | --- | --- | --- | --- | --- | --- | --- | --- | --- | --- | --- | --- | --- | --- | --- | --- | --- | --- | --- | --- | --- | --- | --- |
|  |  | **N** | **T1** | **T2** | **T3** | **N** | **T1** | **T2** | **T3** | **N** | **T1** | **T2** | **T3** | **N** | **T1** | **T2** | **T3** | **N** | **T1** | **T2** | **T3** | **N** | **T1** | **T2** | **T3** | **N** | **T1** | **T2** | **N** | **T1** | **T2** |
| **SH** | **N** | 1.00 |  |  |  |  |  |  |  |  |  |  |  |  |  |  |  |  |  |  |  |  |  |  |  |  |  |  |  |  |  |
|  | **T1** |  | 1.00 |  |  |  |  |  |  |  |  |  |  |  |  |  |  |  |  |  |  |  |  |  |  |  |  |  |  |  |  |
|  | **T2** |  |  | 1.00 |  |  |  |  |  |  |  |  |  |  |  |  |  |  |  |  |  |  |  |  |  |  |  |  |  |  |  |
|  | **T3** |  |  |  | 1.00 |  |  |  |  |  |  |  |  |  |  |  |  |  |  |  |  |  |  |  |  |  |  |  |  |  |  |
| **MRL** | **N** | 0.60** |  |  |  | 1.00 |  |  |  |  |  |  |  |  |  |  |  |  |  |  |  |  |  |  |  |  |  |  |  |  |  |
|  | **T1** |  | 0.68** |  |  |  | 1.00 |  |  |  |  |  |  |  |  |  |  |  |  |  |  |  |  |  |  |  |  |  |  |  |  |
|  | **T2** |  |  | 0.80** |  |  |  | 1.00 |  |  |  |  |  |  |  |  |  |  |  |  |  |  |  |  |  |  |  |  |  |  |  |
|  | **T3** |  |  |  | 0.79** |  |  |  | 1.00 |  |  |  |  |  |  |  |  |  |  |  |  |  |  |  |  |  |  |  |  |  |  |
| **SDW** | **N** | 0.02 |  |  |  | -0.05 |  |  |  | 1.00 |  |  |  |  |  |  |  |  |  |  |  |  |  |  |  |  |  |  |  |  |  |
|  | **T1** |  | 0.34** |  |  |  | 0.16** |  |  |  | 1.00 |  |  |  |  |  |  |  |  |  |  |  |  |  |  |  |  |  |  |  |  |
|  | **T2** |  |  | 0.09 |  |  |  | 0.15** |  |  |  | 1.00 |  |  |  |  |  |  |  |  |  |  |  |  |  |  |  |  |  |  |  |
|  | **T3** |  |  |  | -0.07 |  |  |  | -0.07 |  |  |  | 1.00 |  |  |  |  |  |  |  |  |  |  |  |  |  |  |  |  |  |  |
| **SFW** | **N** | 0.56** |  |  |  | 0.63** |  |  |  | 0.05 |  |  |  | 1.00 |  |  |  |  |  |  |  |  |  |  |  |  |  |  |  |  |  |
|  | **T1** |  | 0.49** |  |  |  | 0.62** |  |  |  | 0.37** |  |  |  | 1.00 |  |  |  |  |  |  |  |  |  |  |  |  |  |  |  |  |
|  | **T2** |  |  | 0.77** |  |  |  | 0.71** |  |  |  | 0.11* |  |  |  | 1.00 |  |  |  |  |  |  |  |  |  |  |  |  |  |  |  |
|  | **T3** |  |  |  | 0.67** |  |  |  | 0.63** |  |  |  | 0.00 |  |  |  | 1.00 |  |  |  |  |  |  |  |  |  |  |  |  |  |  |
| **RDW** | **N** | -0.15** |  |  |  | -0.34** |  |  |  | 0.23** |  |  |  | -0.21** |  |  |  | 1.00 |  |  |  |  |  |  |  |  |  |  |  |  |  |
|  | **T1** |  | -0.42** |  |  |  | -0.43** |  |  |  | 0.21** |  |  |  | -0.24** |  |  |  | 1.00 |  |  |  |  |  |  |  |  |  |  |  |  |
|  | **T2** |  |  | -0.59** |  |  |  | -0.55** |  |  |  | 0.03 |  |  |  | -0.47** |  |  |  | 1.00 |  |  |  |  |  |  |  |  |  |  |  |
|  | **T3** |  |  |  | -0.52** |  |  |  | -0.36** |  |  |  | 0.35** |  |  |  | -0.32** |  |  |  | 1.00 |  |  |  |  |  |  |  |  |  |  |
| **RFW** | **N** | 0.24** |  |  |  | 0.42** |  |  |  | 0.05 |  |  |  | 0.25** |  |  |  | -0.17** |  |  |  | 1.00 |  |  |  |  |  |  |  |  |  |
|  | **T1** |  | 0.42** |  |  |  | 0.33** |  |  |  | 0.40** |  |  |  | 0.51** |  |  |  | -0.11* |  |  |  | 1.00 |  |  |  |  |  |  |  |  |
|  | **T2** |  |  | 0.30** |  |  |  | 0.29** |  |  |  | 0.26** |  |  |  | 0.34** |  |  |  | -0.16** |  |  |  | 1.00 |  |  |  |  |  |  |  |
|  | **T3** |  |  |  | 0.11* |  |  |  | 0.08 |  |  |  | 0.00 |  |  |  | 0.50** |  |  |  | -0.09 |  |  |  | 1.00 |  |  |  |  |  |  |
| **GR** | **N** | 0.23** |  |  |  | 0.31** |  |  |  | 0.03 |  |  |  | 0.15** |  |  |  | -0.24** |  |  |  | 0.09 |  |  |  | 1.00 |  |  |  |  |  |
|  | **T1** |  | 0.23** |  |  |  | 0.24** |  |  |  | 0.13* |  |  |  | 0.13* |  |  |  | -0.08 |  |  |  | 0.08 |  |  |  | 1.00 |  |  |  |  |
|  | **T2** |  |  | 0.23** |  |  |  | 0.24** |  |  |  | -0.06 |  |  |  | 0.16** |  |  |  | -0.12* |  |  |  | 0.12* |  |  |  | 1.00 |  |  |  |
| **GP** | **N** | 0.07 |  |  |  | 0.15** |  |  |  | 0.04 |  |  |  | -0.05 |  |  |  | 0.00 |  |  |  | 0.01 |  |  |  | 0.57** |  |  | 1.00 |  |  |
|  | **T1** |  | 0.11* |  |  |  | 0.01 |  |  |  | 0.20** |  |  |  | 0.00 |  |  |  | 0.05 |  |  |  | 0.08 |  |  |  | 0.55** |  |  | 1.00 |  |
|  | **T2** |  |  | 0.00 |  |  |  | 0.07 |  |  |  | 0.02 |  |  |  | -0.06 |  |  |  | 0.04 |  |  |  | 0.07 |  |  |  | 0.60** |  |  | 1.00 |

* and **means significant at 0.05 and 0.01 probability levels, respectively.

Table S2 Candidate genes prediction of important loci significantly associated with salt tolerance traits

| Trait | Marker | Mapping chromosome | Candidate gene | Function | Biological process/Expression | Species |  |
| --- | --- | --- | --- | --- | --- | --- | --- |
| MRL/SH/SFW | wPt-7187 | 2A | [TraesCSU02G009300](http://plants.ensembl.org/Triticum_aestivum/Gene/Summary?db=core;g=TraesCSU02G009300;tl=0x796mKLhQtWlnpI-21313731-2295993439).1 | calcium ion binding; polysaccharide binding | Unknown | *Triticum aestivum* |  |
|  |  |  | OB01G45130 | peroxidase activity | oxidation-reduction process | *Oryza brachyantha* |  |
|  |  |  |  | calcium ion binding |  |  |  |
|  |  |  |  | oxidoreductase activity |  |  |  |
|  |  |  |  | oxidoreductase activity, acting on NAD(P)H, oxygen as acceptor |  |  |  |
| MRL/SH/SFW | wPt-2185 | 2A | TraesCSU02G212400;  TraesCSU02G212100;  TraesCSU02G212300;  TraesCSU02G212500 | Unknown | Unknown | *Triticum aestivum* |  |
|  |  |  |  |  |  |  |  |
|  |  |  | OB09G21770 | calcium transmembrane transporter activity, phosphorylative mechanism | ion transport | *Oryza brachyantha* |  |
|  |  |  |  | calmodulin binding | calcium ion transport |  |  |
|  |  |  |  | nucleotide binding; ATP binding | calcium ion transmembrane transport |  |  |
|  |  |  | TraesCS2A02G048300; | Unknown | Unknown | *Triticum aestivum* |  |
| RFW | wPt-2087 | 2A | TraesCS2A02G048400;  TraesCS2A02G048500 |  |  |  |  |
|  |  |  |  |  |  |  |  |
|  |  |  | OB06G15330 | ion channel activity | ion transport | *Oryza brachyantha* |  |
|  |  |  |  | voltage-gated potassium channel activity | potassium ion transport |  |  |
|  |  |  |  |  | potassium ion transmembrane transport |  |  |
|  |  |  |  |  | ion transmembrane transport |  |  |
|  |  |  |  |  | transmembrane transport |  |  |
|  |  |  | OGLUM01G14020 | protein binding | response to salt stress | *Oryza glumipatula* |  |
|  |  |  | BRADI_1g21740v3 | metal ion binding;methylated histone binding | regulation of transcription, DNA-templated | *Brachypodium distachyon* |  |
| GP/MRL/RDW/SFW/SDW | wPt-5870 | 3B | TraesCS3B03G0601400LC.1 | Unknown | Unknown | *Triticum aestivum* |  |
|  |  |  | TraesCS3B03G0602400.1 | Unknown | Unknown |  |  |
|  |  |  | TraesCS3B03G0601500.1 | Unknown | Unknown |  |  |
|  |  |  | TraesCS2D02G378000 | ATP binding;calcium ion binding;polysaccharide binding;protein serine/threonine kinase activity | cell surface receptor signaling pathway; protein phosphorylation | *Triticum aestivum* |  |
|  |  |  | OGLUM09G15140 | transmembrane transporter activity | sodium ion transport | *Oryza glumipatula* |  |
|  |  |  |  | transporter activity | transmembrane transport |  |  |
|  |  |  | AET2Gv20055100 | solute:proton antiporter activity | transmembrane transport | *Aegilops tauschii* |  |
|  |  |  | OBART09G14500 | transmembrane transporter activity | sodium ion transport | *Oryza barthii* |  |
|  |  |  |  | transporter activity | transmembrane transport |  |  |
|  |  |  | TRITD3Bv1G214530 | transmembrane transporter activity | transmembrane transport | *Triticum turgidum* |  |
|  |  |  | OB01G34980 | hydrolase activity, acting on ester bonds | response to salt stress；salinity response；response to ionic osmotic stress | *Oryza brachyantha* |  |
|  |  |  | TRIDC2AG075190 | metal ion binding | metal ion transport | *Triticum dicoccoides* |  |
|  |  |  | BGIOSGA031000 | transmembrane transporter activity | sodium ion transport | *Oryza sativa Indica Group* |  |
|  |  |  | ORGLA09G0111500 | transmembrane transporter activity | sodium ion transport | *Oryza glaberrima* |  |
| GR/MRL/RDW/SFW/SDW | wPt-3620 | 3B | TraesCS3B03G0301100LC.1 | Unknown | Unknown | *Triticum aestivum* |  |
|  |  |  | TraesCS3B03G0301400LC.1 | Unknown | Unknown |  |  |
|  |  |  | \| TraesCS3B02G134500.1 \| \| --- \| | Unknown | Unknown |  |  |
|  |  |  | TRIDC3BG068060 | Unknown | Unknown | [*Triticum dicoccoides*](http://plants.ensembl.org/Triticum_dicoccoides/Info/Index?db=core;g=TRIDC3BG068060;r=2A:21569206-21569947;tl=M718boot3McO8kry-21313843-2296000265) |  |
|  |  |  | BRADI_4g16243v3 | zinc ion binding;metal ion binding | regulation of transcription, DNA-templated | *Brachypodium distachyon* |  |
| GP/RDW/SFW | wPt-666008 | 3B | TraesCS3B03G0143800LC | Unknown | Unknown | *Triticum aestivum* |  |
|  |  |  | TraesCS3B03G1233300LC.1 | Unknown | Unknown |  |  |
|  |  |  | TraesCS1D03G0958900LC |  |  | *Triticum aestivum* |  |
|  |  |  | ORGLA04G0060900 | magnesium ion binding;metal ion binding |  | *Oryza glaberrima* |  |
